# Isolation of a Virulent *Clostridium perfringens* Strain from *Elaphurus davidianus* and Characterization by Whole-Genome Sequence Analysis

**DOI:** 10.3390/cimb46070427

**Published:** 2024-07-08

**Authors:** Zhao Zhang, Xiao Wang, Siyuan Li, Yuhang Fu, Yan Li, Shah Nawaz, Jing Chen, Guoxiang Yang, Jiakui Li, Daoliang Shi

**Affiliations:** 1 College of Veterinary Medicine, Huazhong Agricultural University, Wuhan 430070, China; 2 Hubei Provincial Wildlife Rescue Center, Wuhan 430070, China; 3 Department of Forestry Ecology, Hubei Ecology Polytechnic College, Wuhan 430070, China

**Keywords:** *Elaphurus davidianus*, *Clostridium perfringens*, whole-genome sequencing analysis, comparative genome

## Abstract

*Clostridium perfringens* (*C. perfringens*) is an important veterinary pathogen and a noteworthy threat to human and animal health. Recently, there has been a significant rise in the number of moose fatalities caused by this rare, endemic species in China. Currently, there is an increasing trend in conducting whole-genome analysis of *C. perfringens* strains originating from pigs and chickens, whereas fewer studies have been undertaken on *Elaphurus davidianus*-originating strains at the whole-genome level. Our laboratory has identified and isolated five *C. perfringens* type A from affected *Elaphurus davidianus.* The current study identified the most potent strain of *C. perfringens,* which originated from *Elaphurus davidianus*, and sequenced its genome to reveal virulence genes and pathogenicity. Our findings show that strain CX1-4 exhibits the highest levels of phospholipase activity, hemolytic activity, and mouse toxicity compared to the other four isolated *C. perfringens* type A strains. The chromosome sequence length of the CX1-4 strain was found to be 3,355,389 bp by complete genome sequencing. The current study unveils the genomic characteristics of *C. perfringens* type A originating from *Elaphurus davidianus*. It provides a core foundation for further investigation regarding the prevention and treatment of such infectious diseases in *Elaphurus davidianus*.

## 1. Introduction

The *Elaphurus davidianus* is an herbivorous species endemic to China. Currently, only four primary *Elaphurus davidianus* populations exist and rely on sustainable reproduction: the Wubang Temple population, Nanhaizi population, Dafeng population, and Shishou Rewilding population. Notably, as of September 2020, the total *Elaphurus davidianus* population was more than 8000 throughout China. *Elaphurus davidianus* are crucial to the integrity of wetland and plain ecosystems. Protecting these animals and fostering their healthy propagation is an essential task. Such efforts contribute to developing robust and complete ecosystems and biological chains [1,2].

*Clostridium perfringens* (*C. perfringens*), called *Clostridium welchii*, is a zoonotic pathogen typically found in the gastrointestinal tract and soil [3,4]. It is a Gram-positive bacterium that cannot grow in oxygen, thus categorized as an anaerobic organism [4]. If the equilibrium of intestinal microbiota is disturbed, or resulting from any external environmental factors, stress could trigger systemic and intestinal illnesses in humans and animals, posing a significant risk to their health [5,6,7]. Moreover, *C. perfringen* can produce more than twenty toxins [8,9]. Among them, significant toxins are alpha, beta, epsilon, Iota, enterotoxins (CPE), and necrotizing B-like (NetB) toxins [10]. These toxins could lead to wound infections, gas gangrene, human foodborne illnesses, and enterotoxaemia [5]. In a study by Shymaa Moustafa and colleagues, 200 lamb fecal samples were collected from the Qalyubia and Menofa governorates. The prevalence of *C. perfringens*, a gas-producing capsulated anaerobe, was 51.5%. The highest prevalence rate was for type C [11]. *C. perfringens* strains have been categorized into seven toxin types (A–G) based on the toxins produced [9,12]. Their functions have been investigated [3,13,14]. *C. perfringens* produces a variety of toxins, the synthesis of which is stringently regulated by specific genetic control systems [15]. The prevention and control of *C. perfringens* pose a significant challenge due to the intricate diversity of toxin types and a vast variety of host species, combined with inadequate protection by commercial vaccines [16].

Whole-genome sequencing technology is a vital tool for uncovering the genomic characteristics of natural organisms. The resulting genomic information could be widely utilized in clinical practice for detecting and diagnosing pathogenic microorganisms, swiftly identifying pathogen characteristics, monitoring outbreaks, tracing the epidemiology of diseases, and developing new vaccines. Recently, high-throughput sequencing technologies have been successfully applied to analyze microorganisms [17,18,19,20]. Genome analysis of *C. perfringens* in 88 broiler chickens was conducted [21]. The genome sequencing of 48 representative isolates from each pig farm revealed that *Clostridium perfringens* possesses various antimicrobial drug resistance and toxin genes [22]. A report on using whole-genome sequencing to perform genomic and pathogenicity studies on *C. perfringens* from *Elaphurus davidianus* is lacking. So, a highly virulent strain of *Elaphurus davidianus*-originated *C. perfringens* was isolated through screening, and its genomic features and pathogenic genes were analyzed through whole-genome sequencing.

## 2. Materials and Methods

### 2.1. Source of Bacteria and Resuscitating Bacteria

In January 2021, within the Shishou National Nature Reserve for *Elaphurus davidianus* in Hubei Province, China, multiple *Elaphurus davidianus* were found deceased. From the collected tissues and fecal samples of these animals, we successfully isolated and identified five distinct strains of *Clostridium perfringens* with varying levels of toxicity. The stored bacterial suspension was retrieved from a −80 °C freezer. A total of 100 µL of this suspension was added to a reinforced Clostridium liquid medium and then incubated at 42 °C with shaking at 180 rpm for 3 h for revival. Next, 100 µL of the revived culture’s supernatant was spread onto Tryptone Sulfite Cycloserine (TSC) agar plates. These plates were then placed in an anaerobic bag and incubated at 42 °C for 18–24 h. Black, round, singular colonies were picked for Gram staining. A single colony was then streaked onto reinforced Clostridium agar plates and incubated anaerobically at 42 °C for 18–24 h. After purifying for three generations, a single colony was inoculated into the reinforced *Clostridium* liquid medium and incubated on a shaker at 42 °C and 180 rpm for 16 h.

### 2.2. Assay of Phospholipase C Clostridium (PLC) Activity

A total of 100 µL of pre-isolated bacterial solution was incubated for 2 h and transferred to a 96-well plate. As for the control, the same volume of normal saline was used. After centrifugation of the yolk emulsion, the supernatant with saline was diluted at a ratio of 1:10. Then, 100 µL of this diluted solution was mixed thoroughly in each well. Lastly, they were incubated at 37 °C for 1 h, and the OD620 readings were measured using an enzyme marker.

### 2.3. Hemolytic Activity Assay

An appropriate amount of 2% erythrocyte suspension using PBS was prepared. Then, 100 µL of the bacterial supernatant, after a 4-h incubation, was poured into 96-well plates. Equal volumes of erythrocyte lysate and PBS were taken as positive and negative controls, respectively. Finally, 100 µL of 2% erythrocyte suspension was mixed gently into each well. After one hour of incubation at 37 °C, an enzyme marker was used to determine the OD_540_ reading of each well. The hemolysis rate was calculated using the following formula: Hemolysis Rate = (Sample OD_540_ − Negative OD_540_)/(Positive OD_540_ − Negative OD_540_).

### 2.4. Mouse LD_50_ Test

We selected a representative isolate for the mouse LD_50_ assay by combining the results of the PLC activity and the hemolytic activity assays. Purified single colonies for over three generations were inoculated in a *Clostridium perfringens* medium for 5 h. The bacterial solution was diluted into five gradients by 2-fold dilution, and each gradient was inoculated into five mice, 6–8-week-old Kunming mice, at a dose of 0.5 mL/mice. In addition, a blank control was set up, and saline was injected intraperitoneally at a dose of 0.5 mL/mice. The inoculated mice were kept in the same environment. The morbidity and mortality were observed continuously for one week.

### 2.5. Whole-Genome Sequencing Analysis of the Most Virulent Isolates

Genomic DNA was extracted to facilitate further genomic analysis of the most potent strain, and an online library was configured using ONT kits. We used unicycler software to assemble the filtered reads, first with highly accurate Illumina data (Q30 > 85%) and then with Nanopore data to join the high-quality contigs into a completed graph. Finally, Pilon (1.23) software was used, and the assembled genomes were further corrected for errors using the second-generation data. The final genome with higher accuracy was obtained. Prokka (1.12) was employed to identify coding genes within the assembled genomes. Genome sequencing depth, GC distribution, and genome structure annotation were combined to create genome circles using the R package circlize. The presence of gene islands in the genome was also predicted using the IslandViewer 4 website (http://www.pathogenomics.sfu.ca/islandviewer/, accessed on 1 August 2021). A pseudo-finder was also utilized to identify pseudogene candidates from the annotated GenBank files of bacterial and archaeal genomes. A BLAST+(2.5.0+) comparison was conducted by using anticipated gene sequences with functional databases, including COG (https://www.ncbi.nlm.nih.gov/COG/, accessed on 1 September 2021) and KEGG (https://www.kegg.jp/kegg/, accessed on 1 September 2021). Afterward, the entire genome sequencing data of the highly virulent strains were submitted to the VFDB database (http://www.mgc.ac.cn/VFs/, accessed on 1 February 2022) to predict associated virulence factors.

### 2.6. Comparative Genomic Analysis of the Most Virulent Strains

The genomic sequences of the reference strains were acquired from the NCBI website (https://www.ncbi.nlm.nih.gov/genome/, accessed on 1 April 2022). These strains were obtained from various countries and different species. A comprehensive overview of the collected data is depicted in Table 1.

The annotated genome of the virulent strain was compared with the genomic features of eight reference strains selected from the NCBI genome database, and the standard and different essential features of their genomes were clarified with other strains. The fasta format files of the genomes of the nine strains were submitted to JspeciesWS, and the ANI (https://www.ezbiocloud.net/tools/ani, accessed on 1 May 2022) between the genomes was calculated based on the Blast+ algorithm. The genomic ANI heatmap with ANI values was mapped using TB tool (1_098726) software. Geneious prime software (2021.2) was utilized to compare the whole-genome sequence of the *C. perfringens* CX1-4 strain with the reference strain. The NCBI genome database provided the genome sequences of *C. perfringens* ATCC13124, *C. perfringens* Str.13, and *C. perfringens* SM01, submitted to the VFDB database to complete the annotation of related virulence genes. The annotations for the virulence genes of *C. perfringens* Str.13 and *C. perfringens* SM01 were compared to those of reference strains to analyze the common and specific virulence genes. TB tool software created a Venn diagram of the virulence genes.

### 2.7. Statistical Analysis

SPSS 21 Probit regression analysis calculated the median lethal dose of different strains and the LD_50_ of the isolates on mice. GraphPad Prism (v 7.0) was used to conduct statistical analysis. All values were presented as mean ± SD, and *p*-values (corrected) < 0.05 were considered statistically significant.

## 3. Results

### 3.1. Phospholipase C Activity Assay

**Figure 1:**
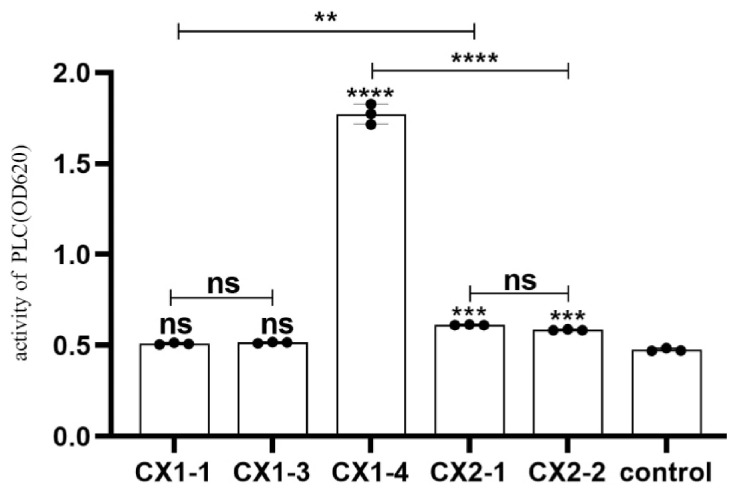
Phospholipase C activity of each isolate, ns: *p* > 0.05; **: *p* ≤ 0.01; ***: *p* ≤ 0.001; ****: *p* ≤ 0.0001.

Among the strains pre-isolated in our laboratory, namely CX1-1, CX1-3, CX1-4, CX2-1, and CX2-2, the PLC activities of CX1-1 and CX1-3 were not significantly different from the control (*p* > 0.05). Conversely, strains CX1-4, CX2-1, and CX2-2 showed significantly higher PLC activity than that of the control (*p* ≤ 0.001), with the CX1-4 strain exhibiting the highest PLC activity (Figure 1).

### 3.2. Determination of Hemolytic Activity

**Figure 2:**
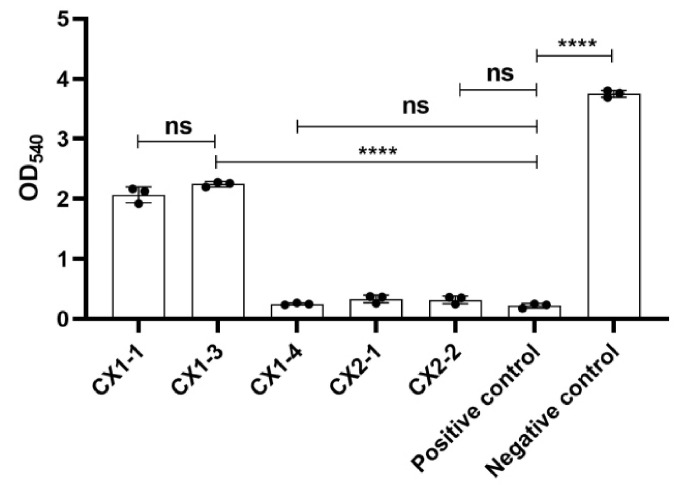
Hemolytic activity of isolates, ns: *p* > 0.05; ****: *p* ≤ 0.0001.

**Figure 3:**
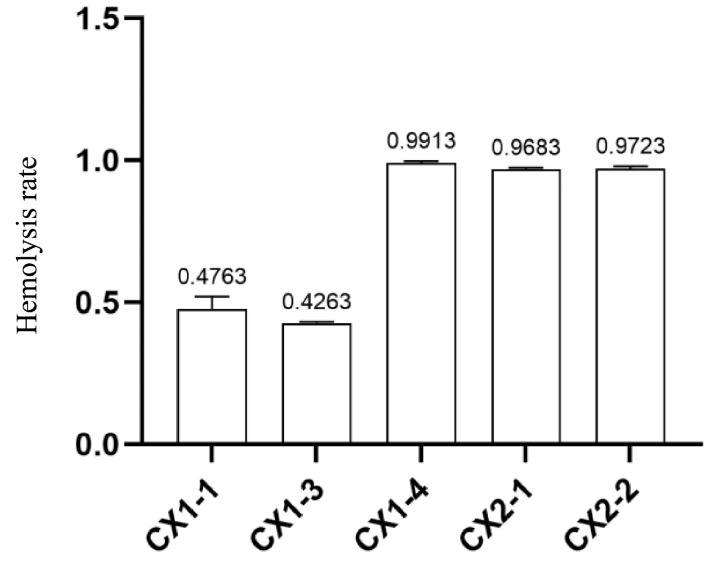
Hemolytic rate of isolates.

The hemolytic activity of all five strains was significantly lower than that of the negative control (*p* ≤ 0.0001). CX1-4, CX2-1, and CX2-2 did not exhibit a significant difference in hemolytic activity compared to the positive control (*p* > 0.05), indicating more significant hemolytic potential (Figure 2). Furthermore, the hemolysis rates of CX1-4, CX2-1, and CX2-2 were above 90%, whereas CX1-1 and CX1-3 exhibited 47.63% and 42.63%, respectively (Figure 3). These findings suggest that CX1-4 possesses the highest hemolytic activity compared to the other isolates.

### 3.3. Pathogenicity Test in Mice

**Figure 4:**
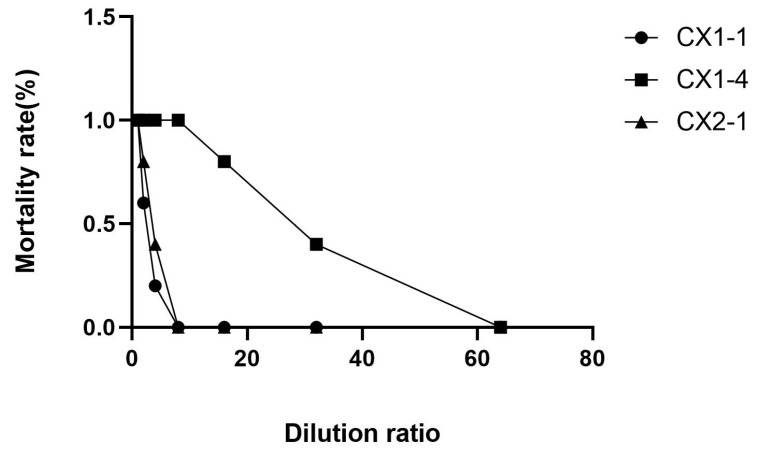
Mortality rate of mice after challenge.

**Figure 5:**
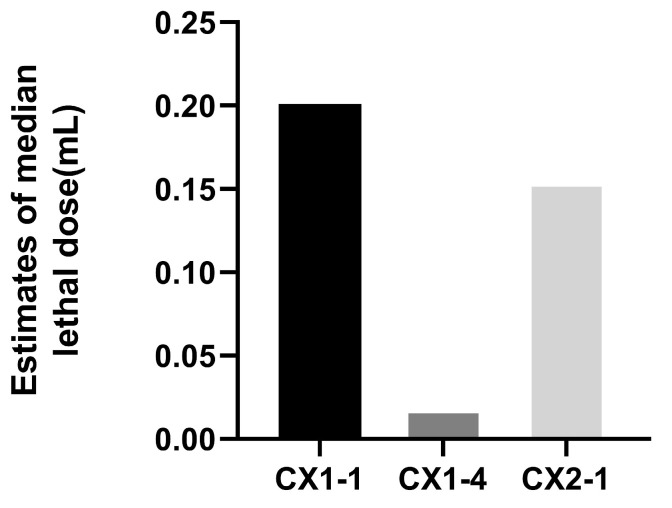
SPSS 21 Probit regression analysis of the half lethal dose of the three.

It was found that the maximum dilution ratio of isolate CX1-4 resulting in mouse death was 2^−5^, whereas, for isolates CX1-1 and CX2-1, the maximum dilution ratio that caused mouse death was 2^−2^, respectively (Figure 4). This suggests that isolate CX1-4 is the most virulent in mice. The SPSS 21 probit regression analysis showed that the half-lethal dose of CX1-4, CX1-1, and CX2-1 was found to be 0.019 mL, 0.201 mL, and 0.155 mL of bacterial solution, respectively. This indicates that the CX1-4 strain is the most toxic (Figure 5).

### 3.4. Whole-Genome Sequencing Analysis of Virulent Strain CX1-4

The CX1-4 strain’s entire genome comprises a chromosome and a cyclic plasmid. The chromosome genome sequence measures 3,355,389 bp in length and has an average GC content of 28.36%. Meanwhile, the cyclic plasmid sequence measures 47,148 bp and has a GC content of 27.33%. Only one sequence greater than 1 kb in length is present in both the chromosome and cyclic plasmid sequences, and no gaps were found between sequences.

In addition, we found that the CX1-4 strain’s chromosomal genome accommodated 3193 genes with a cumulative length of 2,906,888 bp. These genes make up 85.43% of the entire genome’s proportion. Out of this number, 3002 genes with a total length of 2,841,543 bp were gene-encoded, translating to an 83.51% ratio. The genome also comprised 30 rRNA genes, including 10 of 5S, 16S, and 23S; 94 tRNA genes; 66 misc-rna; and 1 tmRNA.

Gene islands and pseudogene four gene islands (GI1, GI2, GI3, and GI4) were predicted to be found in the genome of CX1-4 strains (Table 2). Through KEGG enrichment analysis, we found that genes in the gene island involved DNA mismatch repair protein, tRNA dimethyltransferase, host factor-I protein, pullulanase, and 1,4-alpha-glucan branching enzyme. GO analysis showed that the gene functions in these four gene islands were mainly molecular functions, biological processes, and cellular components. A total of 72 pseudogenes were identified in the genome of strain CX1-4, with a combined sequence length of 36,695 base pairs, corresponding to 1.09% of the entire genome (Table 3). Pseudogenes are enriched in metabolism (23.6%), environmental information processing (0.06%), cellular processes (0.04%), and genetic information processing (0.03%). GO analysis showed that those pseudogenes involve cellular components (44.4%), molecular functions (38.9%), and biological processes (0.29%).

### 3.5. Generic Database Annotation Results

#### 3.5.1. Gene Ontology Classification Results

**Figure 6:**
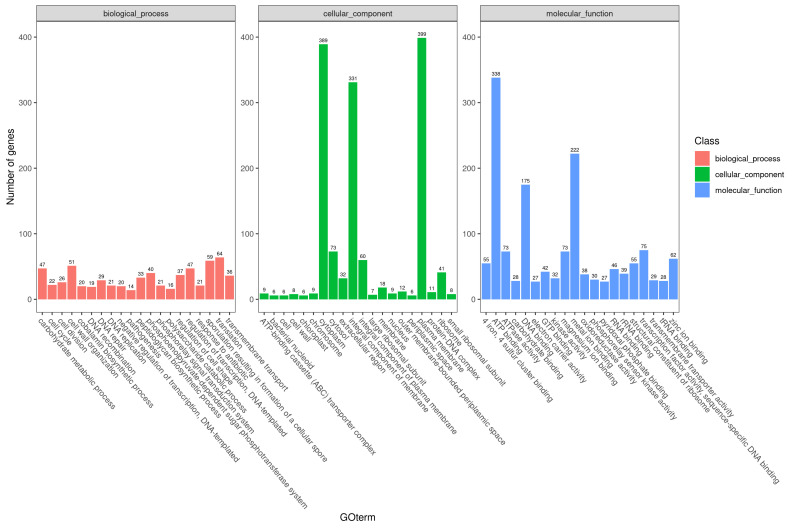
Gene Ontology annotation result classification. The abscissa is the content of each GO (http://geneontology.org/, accessed on 1 October 2021) classification, and the ordinate is the number of genes.

The CX1-4 strain’s genome contains 646, 1440, and 1494 genes associated with cellular components, molecular functions, and biological processes. The quantities for cellular components, molecular functions, and biological processes are 646, 1440, and 1494, respectively. Passive tone and grammatical correctness have been adhered to. In biological processes, 64, 59, and 51 genes were associated with protein translation, spore, and cell wall formation, respectively. The genes enriched in the plasma membrane and cytoplasm and within cellular components were 399, 389, and 389, respectively. Most genes related to ATP, metal ion binding, and DNA polymerization were observed in molecular functions (Figure 6).

#### 3.5.2. KEGG Pathway Classification

**Figure 7:**
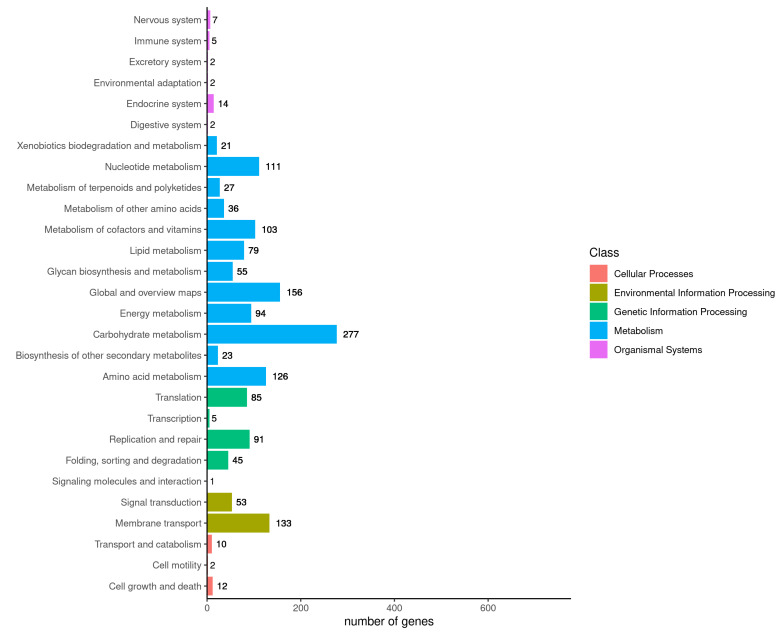
KEGG pathway classification results.

The KEGG metabolic pathways classification indicates that 1557 out of 3193 genes were annotated to 28 KEGG metabolic pathways, which accounts for 48.76% (Figure 7). These 28 metabolic pathways are linked to five cellular activities: cellular processes, environmental information processing, genetic information processing, metabolism, and organismal systems—most genes are involved in metabolic activities, 1108, representing 71.16%. Most of the genes metabolize carbohydrates, nucleotides, enzymes, and vitamins. Additionally, there were 24 genes involved in cellular processes, 186 genes involved in environmental information processing, 227 genes involved in genetic information processing, and 32 genes involved in organismal systems. Of these genes involved in cellular processes, only 1.54% were identified.

#### 3.5.3. COG Classification Results

**Figure 8:**
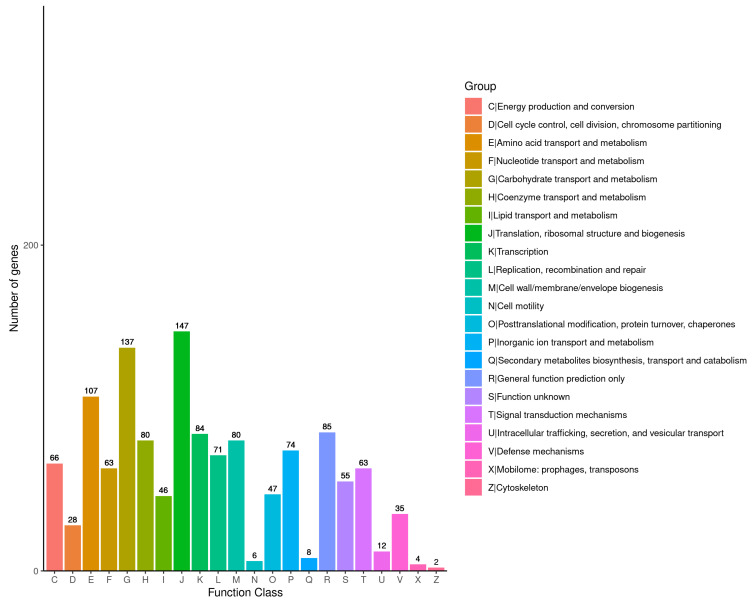
COG classification results.

The results of the COG annotation were divided into 22 categories, with a total of 1300 genes being annotated. Of those, 147 genes (11.31%) were related to translation, ribosome structure, and biosynthesis; 137 genes (10.54%) were connected to transport and carbohydrate metabolism; 107 genes (8.23%) were associated with amino acid transport and metabolism; and only two genes (0.15%) were linked to cytoskeleton-related genes (Figure 8).

#### 3.5.4. VFDB Annotation Results

The VFDB database indicated 13 virulence factors and 20 related genes associated with *C. perfringens* (Table 4). The annotation results of the CX1-4 strain’s virulence factors showed that it carried 18 out of 20 virulence genes, excluding *cpe* and *nagK* virulence genes. This finding further supports the idea that strain CX1-4 is highly virulent.

### 3.6. Results of Comparative Genomic Analysis of Virulent CX1-4 Strain

#### 3.6.1. Comparison of Basic Genomic Features

We found that the genome size of the nine reference strains of *Clostridium perfringens* ranged from 2.9 to 4.0 million base pairs, with a GC content of around 28%. The strains had a gene count between 2694 and 3409, except for strain F262. All strains, save for ATCC13124, had an rRNA count of 30, while ATCC13124 had 24. The tRNA count was approximately 94 for all except F262. Strain SM101 had the highest number of pseudogenes at 116. The Crispr count was about 1 for all strains (Table 5).

#### 3.6.2. Genomic ANI Analysis and Analysis of Genomic Collinearity

**Figure 9:**
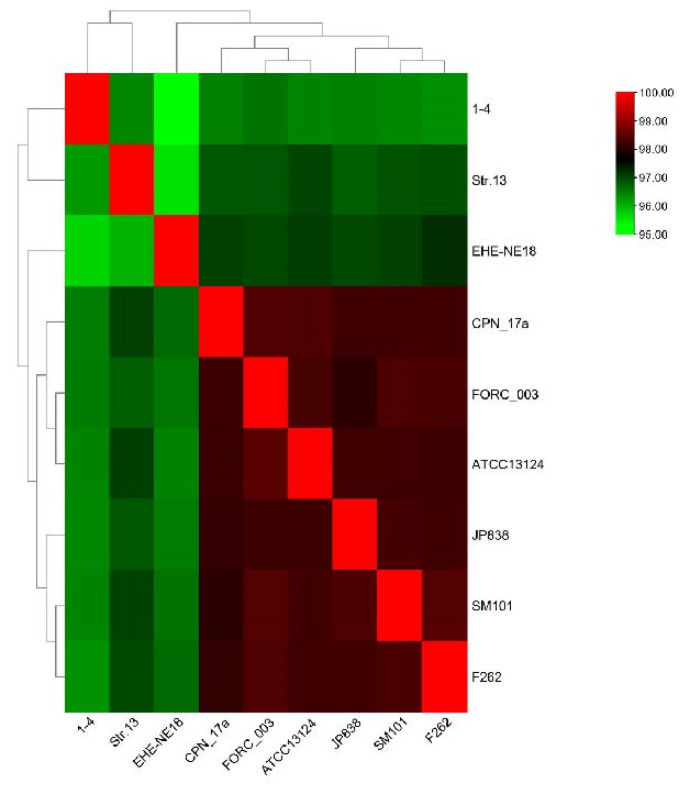
Heatmap of ANI values between groups.

After analyzing the average nucleotide identity (ANI), it was determined that the genomes of the CX1-4 strain and the reference strains displayed ANI values ranging from 95.33% to 96.60%, with an average ANI value of 96.32%. Notably, the lowest ANI value of 95.33% was recorded between the whole genome of CX1-4 and the Australian chicken-derived EHE-NE18 strain, revealing the slightest similarity between these two strains (Figure 9). The average ANI value for A-type strains was 97.30%. ANI values between the CX1-4 strain genome and the reference strains were all lower than the mean value, suggesting a low similarity between the CX1-4 strain and all other A-type reference strains.

**Figure 10:**
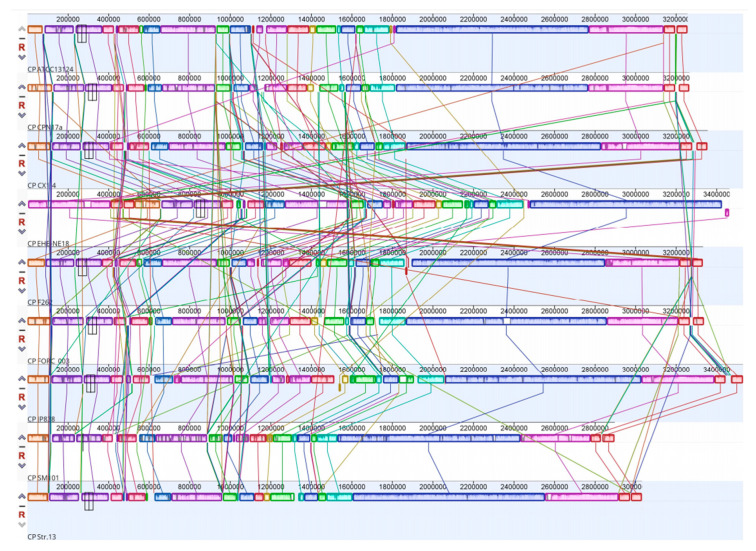
Genome collinearity of 9 strains.

A total of 27 color blocks marking chromosomal homology were identified in nine strains. The strain EHE-NE18 had a significant block inversion mutation at the end of the chromosome compared with other strains. Except for this strain, the remaining strains exhibited strong chromosome homology and consistency in gene order, with minor deletions and insertions. Among the tested strains, it was observed that strain CX1-4 exhibited a significantly higher level of genomic covariance with chromosome F262 than the other strains (Figure 10).

#### 3.6.3. Comparison of Virulence Factors

**Figure 11:**
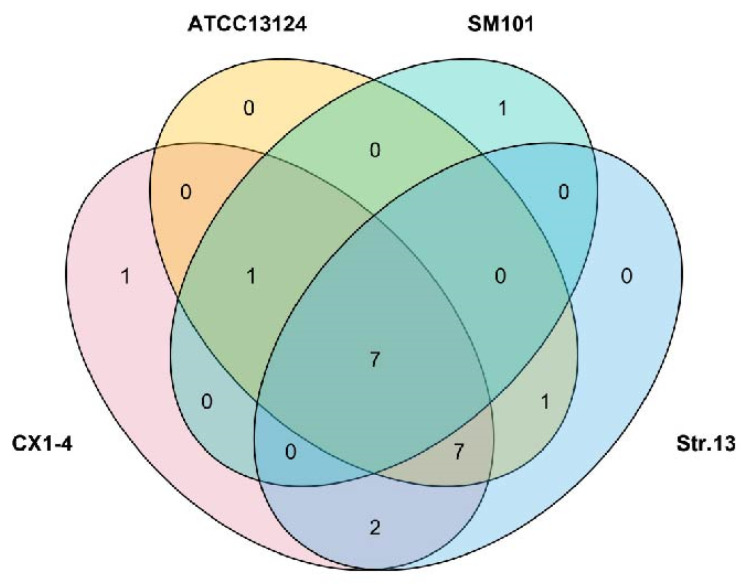
Venn diagram of virulence gene alignment.

The CX1-4 strain had the highest number (18) of virulence genes, elucidating its strong pathogenicity in animal and hemolytic experiments. In contrast, the SM101 strain had the lowest number (9) of virulence genes (Table 6). The seven strains, virS, virR, plc, colA, fbpA/fbp68, cloSI, and Hemolysin, collectively possess seven virulence genes. Among them, CX1-4 and SM101 strains were distinguished by one unique virulence gene each, namely cna and cpe, respectively (Figure 11).

## 4. Discussion

### 4.1. Selection of the Most Virulent Strains

*Clostridium perfringens* (*C. perfringens*), a zoonotic disease, is a persistent threat to animal husbandry and human health. Reports from West Kazakhstan indicate that *C. perfringens* was detected in 30% of tested meat products. [23]. In zoos and wildlife enclosures across India, 30.9% of wild animals were infected with *C. perfringens* [24]. Data from the Centers for Disease Control and Prevention in the United States indicate that up to 1 million cases of foodborne illness are caused by *C. perfringens* annually. This bacterium ranks as the third leading cause of foodborne diseases in the United States, with associated economic losses of up to USD 400 million [25]. Animals with *C. perfringens* disease typically exhibit clinical signs of depression, diarrhea, abdominal distension, and sudden death with pathological lesions like hemorrhage, necrosis, villous shedding, parenchymal organ swelling, congestion, and intestinal hemorrhagic mucosa [7,26]. Human infection with *C. perfringens* can lead to necrotizing enteritis [27]. In the current study, one of the most virulent strains of *Clostridium perfringens* type A was screened based on five isolated and characterized strains in our laboratory. The PLC activity assay demonstrated that all five isolates displayed substantial PLC activity, with the highest activity observed in the *C. perfringens* CX1-4 strain.

*C. perfringens* produces alpha-toxins with PLC and sphingomyelinase activity that hydrolyzes phosphatidylcholine (PC) and sphingomyelin (SM) in the cytoplasmic membrane. This leads to the generation of diacylglycerol (DAG) and ceramide (CER), respectively, destroying cells. The toxins of *C. perfringens* target receptors on the plasma membrane of host cells, subsequently activating intracellular pathways and various cytopathic effects, ultimately leading to cell death [28,29]. Alpha-toxins are sensitive to erythrocytes [30]. The N- and C-termini of the alpha-toxin alone have no hemolytic or lethal activity but are toxic only in combination [31,32]. In this study, it was discovered that five strains of *C. perfringens* exhibited hemolytic activity. Inhibition of the hemolytic activity of *Clostridium perfringens* by baicalin-aluminum significantly reduced intestinal histological damage in broilers infected with *Clostridium perfringens* [33]. Among these strains, *C. perfringens* CX1-4 stood out as having the highest level of hemolytic activity. Notably, this strain was highly virulent in the mouse LD50 assay at 0.019 mL, proving it comparatively more toxic. The findings align with the *C. perfringens* TA-02 LD_50_ from a prior study involving the detection and classification of *C. weissei* in chickens and the assessment of immunogens. These comparative findings suggest that sources of *C. perfringens* type A may exhibit similar levels of virulence.

### 4.2. Whole-Genome Sequencing Analysis of Virulent Strain CX1-4

The essential characteristic of the genome of the *C. perfringens* CX1-4 strain is its consistency with those of the *C. perfringens* genome documented in the database, which indicates no significant structural variations within the genome of the *C. perfringens* CX1-4 strain. The CX1-4 strain’s entire genome comprises a chromosome and a cyclic plasmid. The chromosome genome sequence measures 3,355,389 bp in length and has an average GC content of 28.36%. Gene prediction results revealed 3193 genes within the *C. perfringens* CX1-4 strain genome, consisting of 3002 coding genes, 94 tRNAs, and 30 rRNAs. The genome also consisted of 30 rRNA genes, including 10 each of 5S, 16S, and 23S.

Gene islands are genomic segments derived from laterally transferable genes, often 10–200 kb long, which may contain mobile elements, including phages, integrons, and splice transposons. These elements have been linked to organisms’ pathogenicity, adaptability, and drug resistance [34,35,36,37,38]. Embedding gene islands into the genome is believed to alter the length of mRNA isoforms of “host” genes, thereby enhancing transcriptome diversity [39,40]. In the current study, we identified four gene islands, ranging from 6689 to 11,850 base pairs, that could be associated with the pathogenicity of *C. perfringens* CX1-4 strains. According to KEGG enrichment analysis, genes in the gene island involved DNA mismatch repair protein, tRNA dimethyltransferase, host factor-I protein, pullulanase, and 1,4-alpha-glucan branching enzyme. GO analysis showed that the gene functions in these four gene islands were mainly molecular functions, biological processes, and cellular components. Host factor-I protein is a protein that plays a role in the infection process of a pathogen within its host organism. Bacteriophage λ harnesses the host’s integration host factor protein from *Escherichia coli* to infect its target. Host factor protein also aids in chromosomal compaction and replication initiation, participates in the assembly of higher-order nucleoprotein complexes necessary for site-specific recombination events, and contributes to the transcriptional regulation of specific genes [37,41,42]. Pseudogenes, defined as genome regions containing defective copies of genes, were once considered “Junk DNA.” Bacteria use pseudogenization as a regulatory mechanism to turn off protein function [43].

Interestingly, we found that pseudogenes of CX1-4 are mainly enriched in the metabolism (23.6%), and most pseudogenes involve molecular functions (38.9%) and cellular components (44.4%). In recent years, more and more pseudogenes have been found to play important biological roles in organisms [44,45,46,47,48,49,50,51]. Pseudogenes of CX1-4 may play an important role in molecular function and cellular components. Objective analysis of these findings will enhance our understanding of the mechanisms underlying virulence in these strains.

The Common Database GO annotation results reveal that the *C. perfringens* CX1-4 genome contains many genes responsible for protein translation, cell wall formation, and spore formation. Previous research indicates that the type of toxin expression in *C. perfringens* is stimulated exclusively during spore formation [52]. Furthermore, Shen observed that spore formation is crucial for CPE production in *C. perfringens* [53]. L-glutamine and sodium phosphate (NaPi) at pH 6.0 and AK (pH 7.0) can induce germination of *C. perfringens* spores encoding CCPE, while amino acids and bicarbonate (pH 7.0) can induce germination of *C. perfringens* spores encoding P-CPE [54,55,56,57,58]. The findings suggest a significant correlation between spore formation and the virulence of *C. perfringens*, thereby proposing the hypothesis that *C. perfringens* CX1-4 exhibits greater pathogenicity when spore formation is vigorous during the biological process. Studies have confirmed the adhesive properties of *C. perfringens* spores to Caco-2 cells [59]. The proteins of the gene encoding CX1-4 were annotated according to the KEGG database to demonstrate the underlying metabolic pathways. A total of 3193 coding genes were classified into 28 KEGG functional categories, which mainly play roles in metabolism, genetic information processing, cellular processes, environmental information processing, and organismal systems. Among them, the most genes are involved in metabolism (71.6%). This suggests that CX1-4 has multiple metabolic pathways and can adapt to environmental conditions through metabolism. The *Clostridium perfringens* genome was found to have most genes involved in carbohydrate and protein metabolism [60].

Furthermore, according to the COG annotations, 47 genes (11.31%) were related to translation, ribosome structure, and biosynthesis; 137 genes (10.54%) were connected to transport and carbohydrate metabolism; 107 genes (8.23%) were associated with amino acid transport and metabolism; and only two genes (0.15%) were linked to cytoskeleton-related genes. The intracellular metabolic activity of *C. perfringens* CX1-4 is also vigorous. The COG database has been designed to classify proteins from completely sequenced genomes based on the orthology concept [61]. COG reflects one-to-many and many-to-many orthogonal relationships and superficial one-to-one relationships. COG is used to predict the function of individual proteins or sets of proteins [62]. The core dataset of the VFDB database contains 575 virulence factors for 32 bacterial species, and its functions include basic information on virulence factors, intragenus comparisons, searches, virulence factor analysis, and database downloads [63]. The VFDB database specializes in the collection of virulence factors that have been identified in bacteria [64,65,66,67,68,69]. The results of the annotation of *C. perfringens* CX1-4 in the VFDB database showed that of the 20 virulence factors in the VFDB database for *C. perfringens*, 18 were carried by *C. perfringens* CX1-4. CPA is the most common *C. perfringens* hemolytic toxin. It is produced by all toxin-type *C. perfringens* strains, but type A *C. perfringens* is usually more capable of producing CPA [33]. NanH and NanI, encoded by the chromosomal toxin genes nanH and nanI, with molecular weights of 43 kDa and 77 kDa, respectively, have neuraminidase activity and can cooperate with CPE to invade organismal cells [70,71]. In comparison, *C. perfringens* JXJA17, a type A isolated by Zeng, carried only 10 virulence-related genes, which explains its stronger virulency during the experiment [72].

The genomic comparison of the *C. perfringens CX1*-4 strain with eight reference strains from the NCBI genome database revealed no significant differences between the *C. perfringens* CX1-4 genome and the reference strains. Phylogenetic trees based on genetic systems can be utilized to elucidate the relationships among different populations [73]. Moreover, the genomic ANI analysis of CX1-4 is consistent with these standard strains at the species level, with the lowest ANI value of 95.33% between *C. perfringens* CX1-4 and EHE-NE18. Average nucleotide identity (ANI) is a class of computational analyses that can define species boundaries for archaea and bacteria [74]. This is the first time that a member of the family Potyviridae has been identified as a member of the species Barleria by ANI [75]. Strain CX1-4 had the highest degree of genomic collinearity with the F262 chromosome genome in the covariance findings, and there may be a remarkable similarity in gene function between them [76]. Strain EHE-NE18 demonstrated the inversion of substantial chromosome genome segments at the end. Mutation was also observed in *C. perfringens* NCTC11144. The findings indicate the existence of major segmental inversions in the genome of *C. perfringens*. Currently, the impact of these inversion variants on phenotype remains unclear [77]. In this study, we compared the virulence genes of CX1-4, *Clostridium perfringens* ATCC 13124, Str.13, and SM101, and the results showed that *C. perfringens* possesses the highest number (18) of virulence genes with a distinct virulence gene, cna. These genetic variations justify the differences in pathogenicity among strains and make the unique genes suitable contenders for vaccine development.

## 5. Conclusions

In this study, we individually measured the PLC activity, hemolytic activity, and median lethal dose of five *Clostridium perfringens* type A strains derived from *Elaphurus davidianus*. CX1-4 was identified as the most virulent among these strains and was selected for further investigation. We conducted a whole-genome analysis of CX1-4 and a comparative analysis with sequences in the database. The results revealed that the chromosome of CX1-4 has a total length of 3,355,389 bp with an average GC content of 28.36%. The chromosome contains 3193 genes with a combined length of 2,906,888 bp, accounting for 85.43% of the entire genome. We predicted four genomic islands and 72 pseudogenes in CX1-4.

Additionally, we identified 18 virulence genes in CX1-4, including Cna, Fibronectin-binding protein, and GroEL. Based on the AHI analysis of the genome, we speculate that the gene structure of the CX1-4 strain is distinct and more recent compared to the reference strains. This research uncovered the genomic traits and potential genetic development of *C. perfringens* type A extracted from *Elaphurus davidianuss*. This lays the groundwork for producing vaccines or medications to prevent and treat *Clostridium perfringens* infections in these animals.

## Figures and Tables

**Table 1:** Basic information of reference strains.

Reference Strain	ATCC13124	Str.13	CPN 17a	JP838	SM101	FORC_003	F262	EHE-NE18
SOURCE	Unknown	Japan	Finland	America	America	Korea	Canada	Australia
HOSTS	Human	NA	Cattle	Dog	NA	NA	Cattle	Chicken
TYPE OF SAMPLE	Gas gangrene tissue	Soil	Feces	Feces	Food	Aquarium water source	Contents of the abomasum	Intestinal contents
GENOTYPE	A	A	NA	A	A	NA	A	NA

Note: NA—unknown.

**Table 2:** Gene island prediction.

GI_ID	seq_ID	Start	End	GI_Length
GI1	assembly_1	1,628,448	1,637,836	9389
GI2	assembly_1	2,129,345	2,140,974	11,630
GI3	assembly_1	2,854,714	2,861,402	6689
GI4	assembly_1	3,283,647	3,295,496	11,850

Note: the first column (GI_ID): the predicted gene island ID; the second column (seq_ID): the sequence ID where the predicted gene island is located; the third column (start) (bp) and the fourth column (end) (bp): the predicted starting and ending positions of the gene island in the genome; and the fifth column (GI_length): the length of the gene island sequence (bp).

**Table 3:** Statistics of pseudogene prediction.

Sequence	Pseudogene Size (bp)	Pseudogene Number	Average Length (bp)	Percentage
ctg-1	36,695	72	509.65	1.09%

Note: pseudogene size: the total length of predicted pseudogene sequences; pseudogene number: the number of predicted pseudogenes; and average length: the average length of predicted pseudogenes. Percentage: the proportion of the genome.

**Table 4:** VFDB database annotation results.

VFclass	Virulence Factors	Related Genes	Results
Adherence	Cna	*cna*	+
	Fibronectin-binding protein	*fbpA*/*fbp68*	+
	GroEL	*groEL*	+
Toxin	Alpha-clostripain	*cloSI*	+
	Alpha-toxin	*plc*	+
	Beta2 toxin	*cpb2*	+
	*C. perfringens* enterotoxin (CPE)	*cpe*	−
	Hemolysin	*Hemolysin*	+
	Kappa-toxin (collagenase)	*colA*	+
	Mu-toxin (neuraminidase)	*nagH*	+
		*nagI*	+
		*nagJ*	+
		*nagK*	−
		*nagL*	+
	Perfringolysin O (theta-toxin/PFO)/botulinolysin/tetanolysin O	*pfoA*	+
	Sialidase	*nanH*	+
		*nanI*	+
		*nanJ*	+
Regulation	VirR/VirS two-component system	*virR*	+
		*virS*	+

Note: VFclass: virulence factor type; virulence factors: virulence factor; related genes: related genes; results: result; +: carries; −: does not carry.

**Table 5:** Basic genome information of isolates and reference strains.

Bacterial Strain	Genome Size	GC Ratio (%)	Number of Genes	CDS Region	tRNA	rRNA	Pseudogenes	Crispr Sequence
CX1-4	3,355,389	28.36	3193	3002	94	30	72	0
ATCC13124	3,256,683	28.38	2976	2855	93	24	27	0
Str.13	3,085,740	28.51	2813	2683	96	30	68	0
SM101	2,960,088	28.23	2694	2566	94	30	116	1
JP838	4,070,811	27.94	3202	3014	92	30	65	0
CPN 17a	3,266,195	28.39	2976	2848	94	30	22	2
FORC_003	3,338,532	28.38	3093	2962	97	30	36	1
F262	3,333,039	28.04	3224	3163	76	30	NA	NA
EHE-NE18	3,463,721	28.27	3409	3278	97	30	60	0

Note: NA—unknown.

**Table 6:** Annotation results of virulence factors of each strain.

	Virulence Factors	Related Genes	CX1-4	ATCC 13124	Str.13	SM101
Adherence	Cna	*cna*	+	−	−	−
	Fibronectin-binding protein	*fbpA*/*fbp68*	+	+	+	+
	GroEL	*groEL*	+	+	+	−
Toxin	Alpha-clostripain	*cloSI*	+	+	+	+
	Alpha-toxin	*plc*	+	+	+	+
	Beta2 toxin	*cpb2*	+	−	+	−
	*C.perfringens* enterotoxin (CPE)	*cpe*	−	−	−	+
	Hemolysin	*Hemolysin*	+	+	+	+
	Kappa-toxin (collagenase)	*colA*	+	+	+	+
	Mu-toxin (neuraminidase)	*nagH*	+	+	+	−
		*nagI*	+	+	+	−
		*nagJ*	+	+	+	−
		*nagK*	−	+	+	−
		*nagL*	+	−	+	−
	Perfringolysin O (theta-toxin/PFO)/botulinolysin/tetanolysin O	*pfoA*	+	+	+	−
	Sialidase	*nanH*	+	+	−	+
		*nanI*	+	+	+	−
		*nanJ*	+	+	+	−
Regulation	VirR/VirS two-component system	*virR*	+	+	+	+
		*virS*	+	+	+	+

## Data Availability

The data presented in this study are openly available in NCBI, reference number PRJNA1056153.

## Abbreviations

ANI	Average nucleotide identity
CDS	Coding DNA sequences
COG	Cluster of orthologous group
CPE	*C. perfringens* enterotoxin
GO	Gene Ontology
KEGG	Kyoto Encyclopedia of Genes and Genomes
LD_50_	Median lethal dose
NCBI	National Center for Biotechnology Information
NetB	Necrotising B-like
OD	Optical density
PLC	Phospholipase C
VFDB	Virulence Factor Database
